# A Review of Studies Involving the Effects of Climate Change on the Energy Consumption for Building Heating and Cooling

**DOI:** 10.3390/ijerph18010040

**Published:** 2020-12-23

**Authors:** Yuanzheng Li, Wenjing Wang, Yating Wang, Yashu Xin, Tian He, Guosong Zhao

**Affiliations:** 1 School of Resources and Environment, Henan University of Economics and Law, Zhengzhou 450046, China; yz_li@huel.edu.cn (Y.L.); xin_yashu@sina.com (Y.X.); 2 Academician Laboratory for Urban and Rural Spatial Data Mining of Henan Province, Zhengzhou 450046, China; 3 State Key Laboratory of Urban and Regional Ecology, Research Center for Eco-Environmental Sciences, Chinese Academy of Sciences, Beijing 100085, China; wjwang_st@rcees.ac.cn; 4 Chengdu Academy of Environmental Sciences, Chengdu 610072, China; wangyt@cdaes.org.cn; 5 School of Water Conservancy and Environment, Zhengzhou University, Zhengzhou 450001, China; het.12b@igsnrr.ac.cn; 6 School of Geography and Information Engineering, China University of Geosciences, Wuhan 430074, China; 7 Institute of Geographic Sciences and Natural Resources Research, Chinese Academy of Sciences, Beijing 100101, China

**Keywords:** climate change, energy consumption, statistical method, physical model method, comprehensive assessment model method, degree days method, urbanization effects

## Abstract

The world is faced with significant climate change, rapid urbanization, massive energy consumption, and tremendous pressure to reduce greenhouse gases. Building heating and cooling is one primary source of energy consumption and anthropogenic carbon dioxide emissions. First, this review presents previous studies that estimate the specific amount of climate change impact on building heating and cooling energy consumption, using the statistical method, physical model method, comprehensive assessment model method, and the combination method of statistical and physical model methods. Then, because the heating and cooling degree days indices can simply and reliably indicate the effects of climate on building heating and cooling energy consumption, previous studies were reviewed from the aspects of heating and cooling degree days indices, regional spatial-temporal variations in degree days and related indices, influencing factors of the spatial distributions of degree days, and the impacts of urbanization on degree days. Finally, several potential key issues or research directions were presented according to the research gaps or fields that need to be studied further in the future, such as developing methods to simply and accurately estimate the specified amounts of climate change impact on building cooling and heating energy consumption; using more effective methods to analyze the daytime, nighttime, and all-day spatial-temporal changes in different seasons in the past and future under various environment contexts by considering not only the air temperature but also the relative humidity, solar radiation, population, etc., and further exploring the corresponding more kinds of driving forces, including the various remotely sensed indices, albedo, nighttime light intensity, etc.; estimating the daytime, nighttime, and all-day impacts of urbanization on heating degree days (HDDs), cooling degree days (CDDs), and their sum (HDDs + CDDs) for vast cities in different environmental contexts at the station site, city, regional and global scales; producing and sharing of the related datasets; and analyzing the subsequent effects induced by climate change on the energy consumption for building heating and cooling, etc.

## 1. Introduction

Buildings are among the sectors with the highest energy consumption, accounting for approximately 40% of the total global energy consumption and generating more than 30% of human-made carbon dioxide emissions [1]. A considerable part of this energy consumption is used in space heating and cooling. Heating, ventilation and cooling account for 35% of primary energy use in America, while a similar level will be reached within five years in China [2]. Meanwhile, near-surface temperatures have increased significantly in most parts of the globe over the past 100 years, especially in recent decades [3]. During the 62 years from 1951 to 2012, the temperature increased by 0.12 °C every 10 years, which was 1.88 times the increase observed since 1880 [3]. Moreover, in recent decades, rapid and intensive urbanization has occurred worldwide, especially in Asia and Africa [4]. In 2018, 55 percent of the world’s population lived in cities. By 2050, the proportion will rise to 68% [4]. Rapid and high-intensity urbanization can lead to significant changes in urban air temperatures, relative humidity, wind speeds and other climatic factors due to the increase in impervious urban surfaces, the decrease in vegetations and water bodies, the influxes of population and the increase in human activity intensities. The median decline and increase in global urban building heating and cooling energy consumption caused by urban heat islands caused by urbanization were 18.7% and 19.0%, respectively [5]. However, the effects varied significantly among different cities [5]. The heating decrease rates were 3–45%, and the cooling increase rates were 10–120% [5]. In the future, urban climate change will have a more significant impact on energy consumption with the rapid progression of global urbanization, the increase in ownership and utilization rates of air conditioning [2,6], the improvements in people’s economic conditions [7], the popularity of glass facade buildings [8], and so on. The urban heat island effect, for example, can cause consequences that are not only related to the energy consumption for heating and cooling but also to the worsening of comfort conditions in buildings (working and educational spaces firstly) and to the increase of the stress principally in the most sensible part of the population (such as old people) in many time periods and places with hot climate [9,10,11]. The greater energy demand for cooling is caused by the increased heat stress may well present a growing threat to human health [11,12] and economic productivity globally [11,13].

It is of great theoretical and practical value to analyze the influences of global, regional or urban climate change on building heating and cooling energy consumption. Therefore, numerous previous studies have been carried out in this research field. However, relevant reviews are quite scarce. Thus, this paper aimed to review the related previous studies in terms of the adopted methods, including the statistical method, physical model method, comprehensive assessment model method, the combination method of statistical and physical model methods, and degree days method; finally, several potential key issues and research directions were proposed.

## 2. Specific Amounts Estimation of Climate Change Influences on Building Heating and Cooling Energy Consumption

Many studies have been carried out on estimating the specific amounts of influences of global, regional or urban climate change on building heating and cooling energy consumption in the past or future. Research methods have mainly included statistical method, physical model method, comprehensive assessment model method, and the combination of the statistical and physical model methods (Table 1).

### 2.1. Statistical Method

**Figure 1:**
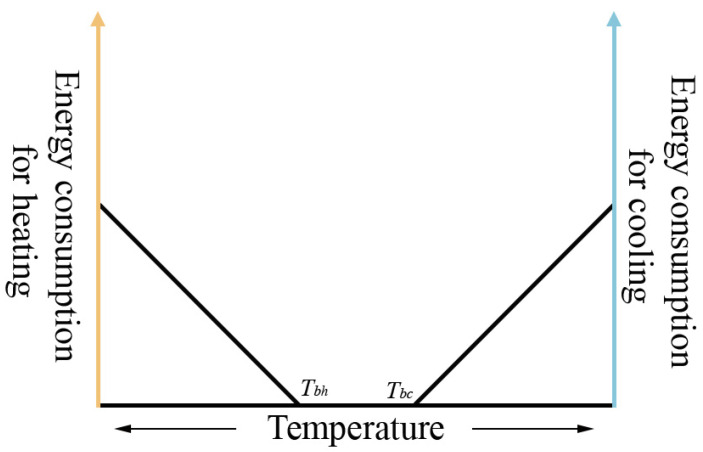
Schematic diagram of the relationship between energy consumption and temperature for heating and cooling. $T_{bh}$ and $T_{bc}$ are the baseline temperature for heating and cooling, respectively.

The statistical method aimed to establish the relationship between the meteorological factors and the total energy consumption of a single building [14,15], energy consumption that has overcome the social and economic impacts by the de-trending method [16,17,18], or the components of energy consumption caused by seasonal changes obtained through multiplicative decomposition [19] for a region or city (Figure 1); or build the relationship between the total energy consumption [20,21], energy consumption per capita [6,22], energy consumption per household [6], or energy consumption per unit of gross domestic product (GDP) [23] of a city or region, and meteorological, social or economic factors. These meteorological factors can be classified as air temperature driven type, non-air temperature driven type, and compound driven types. The air temperature driven factors include hourly temperature [14], daily maximum temperature [14,16], minimum temperature [14], and mean temperature [14,18,20,23], monthly mean temperature [6], and degree days [14,17,24], degree-hours [25], degree-minutes [24], number of days during the heating period [22], power function of degree days [22] calculated based on temperature, etc. Non-air temperature driven factors mainly include the total solar radiation [24], average relative humidity [6,24], total precipitation [16,24], days of precipitation [6], average wind speed [6,24], average atmospheric pressure [24], and so on. Compound driven type factors have also been proposed, such as a modified air temperature index considering the air temperature, urban heat island effect, humidity effect and cumulative effect [26]. Social or economic factors mainly include GDP per capita [17,22], total GDP [21], income per capita [22,25], urbanization rate [22], population [21], number of employees [25], energy price [21], and so on. Methods used to establish the relationship between energy consumption and its influencing factors mainly include simple regression analysis [6,16,19,21,22,25], subsection regression analysis [14], econometric model [15,24], partial correlation analysis [16], bivariate correlation analysis [16], Spearman rank correlation analysis [27], polynomial fitting method [20,28], and so on.

The statistical method has some limitations. First, this method usually requires building heating and cooling energy consumption records with a long historical span and the corresponding driving factors of the same spatial and time caliber [18,19,20,23,37]. This condition is not easy to satisfy. First, the data itself are sometimes not always readily available. For example, since 2005, China’s electricity data have been stated confidential data [46]. Second, the energy consumption data for building heating and cooling are often mixed with these data for other uses. Third, the statistical caliber of the obtained building heating and cooling energy consumption data may not be consistent with the driving factors. Therefore, the relationship between building heating and cooling energy consumption and climate factors may be weakened largely by mixing together data from different backgrounds or time periods due to different driving mechanisms. For example, it is very difficult to separate urban and rural energy consumption data in China [46]. Second, the energy consumption data of building heating and cooling are not only affected by climate, but also by economic, social and technological factors. On this basis, previous studies have usually adopted energy consumption per capita, per household or per unit GDP rather than total energy consumption, or used de-trending methods to eliminate the interference of other factors. However, it is difficult to completely eliminate the interferences of non-climatic factors because there are too many factors and their interactions are complex, and the tendencies of some social, economic and climatic factors themselves. Third, the spatial and temporal resolutions of the research results obtained based on the statistical method are low [5].

### 2.2. Model Method

#### 2.2.1. Physical Model Method

The physical model method has commonly used software to simulate the heating and cooling energy consumption of buildings under past, present and future climate or building parameters, including TRNSYS [32,33,40], EnergyPlus [30,47,48,49,50], thermal analysis system [51], VisualDOE [29,39], IES-VE [52], etc. According to different simulated building objects, this method can be further divided into the prototype method that simulated some artificial set buildings, that did not exist in reality but could realistically represent the main building types [30,31,32,33,38,47,50], and the sample method that simulates highly representative buildings that existed in the real world [51]. The parameters required in the model to represent past and present states were derived from actual observations or statistics [30,31,51]. Future meteorological and building parameters were mostly derived from numerical model simulation and relevant planning respectively [31,51].

In addition, previous studies used physical models to analyze the impacts of urbanization on the energy consumption for building heating and cooling by comparting the differences in energy consumption under different urban and rural meteorological parameters derived from meteorological observation records, including temperature, relative humidity, wind speed, dew point temperature, etc. [32,33,47]. Moreover, the local microclimate changes under different ameliorative measures can be simulated by using the urban microclimate simulation software, such as ENVI-Met. Then, these meteorological parameters were input into the energy consumption software to obtain the corresponding amounts of energy consumption, and the impacts of different ameliorative measures on energy consumption were further analyzed [52].

This method requires very detailed building and environmental parameters, such as the orientation, position and size of doors and windows, lighting inside the building, number of people, building materials, other energy consumption terminals, etc. (Table 2) [30,50]. This method can simulate energy consumption up to the hour scale, spends on a large amount of computational time, requires a high level of professional knowledge, and is mainly suitable for a single building or local scale studies [32,33,47,49,50]. Based on the simulation of a typical single building, a few studies have extended the research to the regional scale by combining the investigation data of different types of single buildings and their energy consumption values [30,31].

#### 2.2.2. Comprehensive Assessment Model Method

In this method, some key parameters affecting the heating and cooling energy consumption of buildings were selected instead of the overly detailed building parameters for rough simulation [34,35,36] or calculation [7,37]. Some unknown parameters were roughly and simply set to a fixed value. This method is usually used to simulate energy consumption at the year [7,35] or month [37] scale, and is suitable for regional [35,37] or global scale [7].

### 2.3. Combination Method of the Statistical and Model Methods

In this method, firstly, the building heating and cooling energy consumption simulated by the physical model was taken as the actual energy consumption. Secondly, the relationship between the simulated energy consumption and climate factors was established. Finally, the impacts of future climate change on energy consumption were predicted [29,38,39,40]. This method can estimate specific amounts of influences of climate change on building heating and cooling energy consumption in a single building without using actual energy consumption data. Moreover, it can alleviate the problems of time-consuming and complex operation of the physical model method to some extent, but it still cannot avoid the inherent defects of the physical model method.

## 3. Influences of Climate Change on the Energy Consumption of Building Heating and Cooling Based on the Degree Days Method

Building heating and cooling energy consumption is generally assumed to be proportional to the differences between indoor and outdoor temperatures. Based on this assumption, the degree days indices, which are closely related to temperature, have become a widely recognized and adopted simple, efficient and reliable indicator to represent the impacts of climate change on the energy consumption of building heating and cooling [43,44,45,53].

### 3.1. Indices Related with Degree Days

From the indicated content aspects, the existing indices of degree days can be mainly divided into three categories: heating degree days (HDDs), cooling degree days (CDDs), and their sum (HDDs + CDDs). HDDs and CDDs refer to the sum of the degrees in which the outdoor average daily temperature is lower than the heating or higher than the cooling reference temperature, calculated within the numbers of days characterizing the heating or cooling period respectively (Equations (1) and (2)) [53]. In addition, some studies have used HDDs + CDDs to indicate the total energy consumption of heating and cooling [54,55]. Other derived indicators have also been applied, including the numbers of days during the heating or cooling period [42,56,57], beginning and ending dates of the heating or cooling period [42,56], and boundary lines of heating areas [56].

(1) $$CDD=\sum_{i=1}^{n}{\left(T_{meani}-T_{bc}\right)}^{+},$$

where $T_{meani}$ is specified as the mean daily air temperature on the ith day during the study period, usually referring to the cooling period; $T_{bc}$ is the baseline temperature for cooling; n is the days in the study period, usually referring to the cooling period; and + means that only positive values can be used.

(2) $$HDD=\sum_{i=1}^{n}{\left(T_{bh}-T_{meani}\right)}^{+},$$

where: the $T_{meani}$ and + have the same meaning in Equation (1); $T_{bh}$ is the baseline temperature for heating, n is the days of the study period, usually referring to the heating period.

From the calculation aspects, these indices can be mainly divided into two categories: temperature-driven and multifactor-driven indices. Temperature-driven indices were calculated based on only air temperature data, mainly including degree days calculated based on quarterly [41], hourly [58,59], daily [26,45], or monthly mean temperatures [60], degree-hours based on hourly temperatures [61], and degree-minutes based on minute temperatures [24]. The setting of the reference temperature was related to the accuracy of indicating the actual energy consumption of such indicators [60,62,63,64]. The reference temperature was mainly determined based on the relationship between actual energy consumption and temperature data [62,63,64,65] or complied with the standards specified by international, national or regional authorities [42,56,58,60,66,67,68]. The multifactor-driven indices not only considered the air temperature, but also other factors closely related to energy consumption. These factors included degree days calculated by the heat index or humidex, both of which were derived from both air temperature and relative humidity [43], CDDs calculated based on wet-bulb temperature [44] or enthalpy which accounts for latent heat as well as sensible heat [69], degree days based on environmental stress index that considered temperature, relative humidity and solar radiation at the same time [43], and an extended version of CDDs calculated based on temperature, including the temperature, specific humidity effects, and residual temperature in recent days [70].

Despite the existence of a variety of degree days indicators, most of the existing studies still adopt temperature driven indicators, which only consider the impacts of temperature on energy consumption and ignore the roles of factors such as relative humidity, solar radiation, etc. [43,46,70]. In addition, due to the lack of long-term detailed time-interval temperature observation data, most studies have adopted the degree days calculated based on the average daily temperature, which was unable to characterize the inter-day detailed changes in energy consumption during all-day period [61].

### 3.2. Spatial-Temporal Changes in Indices Related to Degree-Days

**Figure 2:**
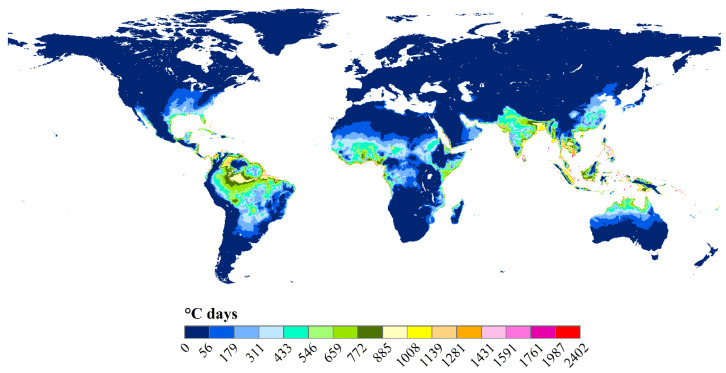
Global cooling degree days accounting for humidity by setting the base temperatures as 18 °C. The data were derived from http://hs.pangaea.de/model/MistryM_2019a/CDD_wetbulb_GeoTIFF.tar.gz [33]. The classification thresholds were determined by the natural break method.

Existing studies have analyzed the spatial-temporal changes in HDDs or CDDs [43,45,53], HDDs + CDDs [55], numbers of days during the heating or cooling period [42,71,72,73], start and end times for heating [42,56], boundary lines for heating areas [56] etc., in the past [43,44,45] or the future [20,55,74] at the city scale [58,75], region [53,76] or global scale (Figure 2) [43,44,45], and have used observational records of sites [45,53], simulation results from climate models [71,77] or reanalysis data [43,44]. Previous studies have mostly been carried out at the site scale and analyzed the spatial-temporal changes in degree days by using site observation data [42,56,64,72,78]. Some studies used the inverse distance interpolation [45], ordinary kriging model [55], co-kriging model [58], regression kriging [41], kriging with external drift scheme [60], and the multiple regression model [79] to interpolate the results on the sites to the surface. Moreover, some previous studies analyzed the interannual variation trends of degree days, by using the Man-Kendall method [42,44,56,80], linear trend analysis [42,78], or a simple comparison of degree days in the historical and future periods [20], etc.

Some deficiencies still exit in this research field. Firstly, previous studies generally analyzed the spatial-temporal changes in all-day HDDs or CDDs in a specific region during the whole heating or cooling period. Comparative studies are lacking in terms of the variation rules between the daytime and nighttime, and among different seasons or regions with different contexts. However, the mechanisms of the degree days are likely to be different under different conditions. Secondly, most of the existing studies have used the degree days calculated by daily temperature, ignoring the influences of natural factors such as relative humidity, solar radiation, wind speed, etc., and social and economic factors such as population and GDP, etc., and also have failed to indicate the inter-day changes in detail. Thirdly, most studies have analyzed the interannual variability of degree days by only calculating the variation rates and their significance. Other research contents or effective analysis methods can be considered in the future. For instance, the future change trends of degree days can be explored using the Hearst exponent calculated by the rescaled range analysis method. Regionalization of degree days changes can be realized using the rotated empirical orthogonal function or cluster method. Both geostatistical analysis and landscape methods can be considered to analyze the spatial-temporal patterns more effectively. Fourthly, the degree days are different in urban, suburban and rural areas. Nevertheless, this issue has not been fully considered by previous studies focusing on the analysis of spatial-temporal changes in a certain area. The spatial resolutions of both climate models and reanalysis data were too crude to fully reveal the above-mentioned differences. The spatial resolutions of the former were 0.11°, approximately 12.5 km [76], or even 0.25–1° [71], while the spatial resolution of the typical reanalysis data from the National Center for Environmental Forecasting was 0.25°. However, most of the studies based on the observation data of stations directly used the data of all stations without distinguishing the types of stations [45], thus leading to a certain level of error. It was truly appreciated that some studies interpolated the observed results at these station points to the regional extent by considering some urbanizing factors. For example, Schatz and Kucharik [41] used the regression kriging or linear regression method to consider the influences of the abundances of the impervious surfaces on the upscaling (from the station point scale to the region space field scale) of HDDs and CDDs in Madison, Wisconsin USA.

### 3.3. Influece Factors of the Spatial Distribution of Degree Days

Mastering the influencing factors of degree days is not only necessary for the regulation of the energy consumption of heating and cooling, but also beneficial to accurately extend the observation results on the station points to the space field of the region. However, such studies are very limited (Table 3). The current study areas were limited to a very small number of places, including Madison, Wisconsin, USA (Table 4) [41], Florence, Italy [68], Andalusia Autonomous Region, Spain [79], Bangladesh [53], Xinjiang, China [80], etc. Previous studies have only analyzed the driving factors of HDDs or CDDs throughout the whole day, ignoring the differences in the mechanisms of degree days between daytime and nighttime, or among different seasons, and have not considered the influencing factors of HDDs + CDDs. Factors that have been considered were limited to latitude, longitude and altitude [53,80], distance to large water bodies [41,79], abundance of impervious surfaces [41,68], large scale atmospheric circulation indices [53], etc. The analysis methods mainly included partial correlation analysis [79], linear regression [53], spatial regression [41], Random Forest (RF) model [53], etc.

### 3.4. Impacts of Urbanization on Degree Days

**Figure 3:**
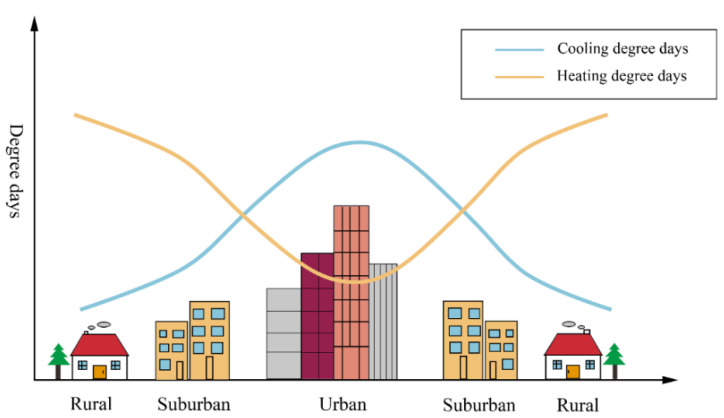
Schematic diagram of the impacts of urbanization on heating and cooling degree days.

To clarify the impacts of urbanization on degree days (Figure 3), existing studies usually simply compare the differences in the HDDs or CDDs calculated based on daily temperature data between urban and suburban or rural stations of a single city [41,81,82,83]. To the best of our knowledge, only Klimenko, et al. [84] compared the differences in the HDDs and CDDs between the urban and suburban stations for several Russian cities.

Existing studies have usually ignored the differences in the influence of urbanization influence on the degree days within cities, among different cities, between the daytime and nighttime, and among different seasons or years, as well as the effects of relative humidity, population, and other factors. Moreover, studies are still lacking which evaluate the urbanization effects on HDDs, CDDs, and HDDs + CDDs for numerous cities under different environmental contexts, using the hourly or even subhourly data, considering not only the air temperature but also the humidity, solar radiation, population, etc., at multiple spatial scales, including station, city, and regional scales.

## 4. Future Directions

Although many studies have been carried out on the impacts of climate change on building heating and cooling energy consumption, there are still some blank areas or research fields that need to be further deepened. Based on the above review, several potential key issues or research directions are proposed.

### 4.1. Estimating the Amounts of Impacts of Climate Change on Building Heating and Cooling Energy Consumption

What are the adaptation possibilities and what can be done to mitigate the impact of climate change on buildings energy requirements? The effort to guarantee high levels of energy efficiency in the building sector is currently recognized as an important goal and it is also promoted by national and international legislation [85]. Simpler and more effective methods are urgently needed to accurately assess the impact of climate change on building cooling and heating energy consumption accurately, especially in regions where it is extremely difficult to obtain and separate data on building heating and cooling energy consumption. These studies may be carried out at different scales, or by considering different elements. For instance, which kinds of design schemes are more energy-saving buildings under various contexts? These potential designs can be obtained by investigating the various functions of the existing buildings, and the ideas of the designers can also be brought into play. Both passive and active strategies can be developed in these designs, including adopting the correct orientation of the buildings, planning the ventilation corridors properly, using more effective materials, taking full advantage of the solar radiation, integrating of new systems fed by solar, wind, geothermal, hydroelectric energy, etc. [85]. Not only the building but also the vegetation and water elements should be considered by using the building energy consumption and urban microclimate simulation software (such as ENVI-Met) together. The amount and pattern of green or water space, vegetation types (forest, shrub, and grass) or species, greening methods (roof greening, horizontal, and vertical greening) and so on can be considered. Similar studies can be carried out at the building block scale. The two- and three-dimensional information about buildings, green spaces, and water bodies in urban and regional areas can be obtained by using various methods, including remote sensing, geographic information technology, field research, viewing the statistical data, and big data technology, etc. Based on the above-mentioned information and the simulation results at the building and building block scale, the amounts of impacts of climate change on building heating and cooling energy consumption can be estimated at the city or region scale.

### 4.2. Spatial-Temporal Changes in Heating and Cooling Degree Days and Their Influencing Factors

Studies focusing on more representative study areas under different contexts, especially at the global scale, should be considered, rather than limiting to a few or some cities or a specified region. Urban, suburban, and rural regions should be treated as separate, rather than being mixed together. Both the intra-annual and inter-annual spatial-temporal changes in degree days should be studied in the HDDs, CDDs, and HDDs + CDDs during not only the all-day periods, but also during the daytime and nighttime in the past and the future. The degree days indices should be calculated based not only on the daily air temperature, but also on the relative humidity, solar radiation, population, etc. Moreover, hourly or even minute-level data should be used at higher density stations. More effective methods should be introduced to more deeply analyze spatial-temporal changes, such as the Hurst exponent method, rotated empirical orthogonal function (REOF) method, geostatistical and landscape analysis, etc.

**Figure 4:**
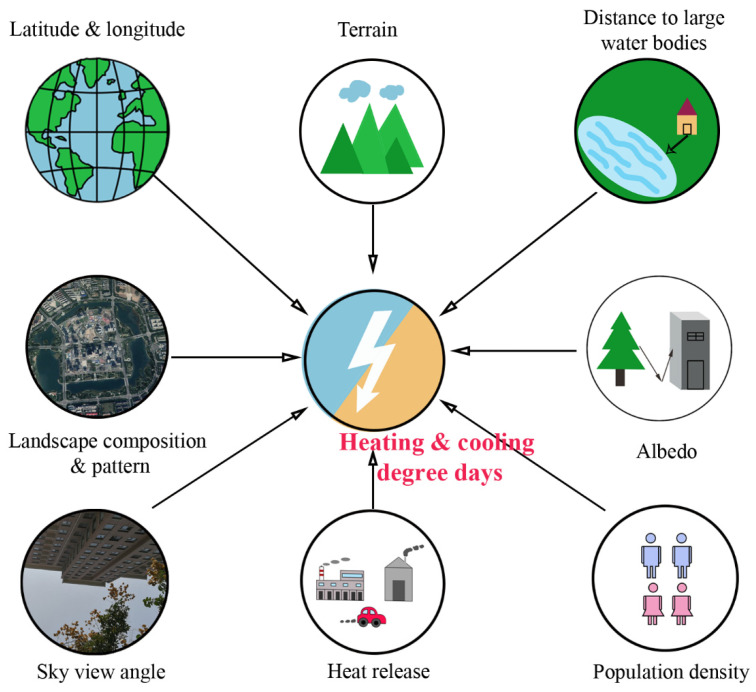
Potential influencing factors of heating and cooling degree days.

Future studies should be carried out on the natural, social, or economic factors influencing the spatial-temporal changes in HDDs, CDDs, and HDDs + CDDs during daytime, nighttime, and all-day in different seasons under different climatic contexts, such as landscape composition and pattern, remote sensed indices of building, vegetation, water or bare soil, albedo, nighttime light intensity, sky view factor, heat release, population density, etc. (Figure 4). In addition, the analysis method of the influencing factors of degree days also needs to be improved. For instance, the scale effects of influencing factors for each degree days index under different conditions should be considered. More effective methods should be introduced, such as the machine learning method.

### 4.3. Impacts of Urbanization on Heating and Cooling Degree Days

It is necessary to accurately estimate the effects of urbanization on the heating and cooling degree day during the daytime, nighttime, and all-day in different seasons and the whole year under different environmental contexts at different scales, which can be possible done by comprehensively utilizing multi-sources data and several methods. These data may include hourly or subhourly meteorological data of high-density stations, land use data in the middle or even high spatial resolution, raw remote sensing data, MODIS data, defense meteorological satellite program (DMSP), and visible infrared imaging radiometer suite (VIIRS) nighttime light data, digital elevation models, spatially discrete population data, big data, etc. These methods may involve station observation methods, geographic information technology, remote sensing method, mathematical statistics, grey correlation, machine learning method, landscape analysis, abrupt climate change detection, homogenization of climate data, etc. It should be noted that a large number of studies have been carried out on the urbanization effects on the air temperature, apparent temperature, and land surface temperature closely related to degree days, which can provide important references for these studies on degree days. For example, previous studies on urban heat islands [86,87] and the contribution of urbanization to temperature change [88,89] can provide important theoretical and methodological references for analyzing the impacts of urbanization on degree days. Based on the land use data, urban and rural stations can be identified. Then, the impacts of urbanization at the site scale can be defined as the differences in the HDDs, CDDs, and HDDs + CDDs, and their corresponding interannual variation rates between the urban and rural stations during the daytime, nighttime, and all-day in each season and the whole year. The HDDs, CDDs, and HDDs + CDDs at the regional scale can be obtained based on their values at the sites, and their relationships with many influencing factors. Finally, the urbanization effects on HDDs, CDDs, and HDDs + CDDs at the regional scale can be derived based on urban and rural areas using land use data.

### 4.4. Production and Share of Related Datasets

Accurate high-resolution datasets are extremely important for understanding the effects of climate change on the energy consumption of building heating and cooling. These datasets may involve weather data (including near-surface temperature, humidity, solar radiation, heating and cooling degree days, etc.), energy data of building heating and cooling (consumed fuel type and quantity), building parameters, land cover/use data, digital elevation data, raw remote sensed data, remote sensed indices, nighttime light data, sky view data, air conditioning usage data, motor vehicle data, population data, gross domestic product per capita, related standards or policies, etc. These datasets should have high spatial and temporal resolutions, consider not only the past but also the future period, distinguish the differences between the urban and rural regions, take into account the heterogeneity within the city, etc. For instance, the heating and cooling degree datasets should consider not only the temperature, but also the humidity, solar radiation, wind speed, population density, etc. in hourly or even minute resolution in the past and the future, produced based on the observed data in the higher density weather stations, taking into account the heterogeneity inside the city or differences among different cities or regions, etc. While producing and sharing these data sets is quite important, multidisciplinary efforts and cross-cutting studies are required in the future.

### 4.5. Subsequent Effects Induced by the Climate Change on the Energy Consumption of Building Heating and Cooling

Climate change can not only affect the energy consumption of building heating and cooling, but also further cause other issues, such as the emissions of carbon dioxide and the release of air pollutants (sulfur dioxide, nitrogen oxide, freon, PM2.5, PM10, etc.), decrease of the comfort level in human settlements, increase of the disease risk among residents, leading to water thermal pollution and further destroying its ecological balance, impeding the increase of economic productivity, etc. All these research fields should be encouraged to be studied in the future.

## 5. Conclusions

This paper aimed to summarize the important progress of previous research involving the effects of climate change on the energy consumption of building heating and cooling, analyze some shortcomings in this research field, and propose several potential research directions. The main findings are as the follows.

(1) Estimating the amounts of impacts of global, regional, or urban climate change on building heating and cooling energy consumption accurately can provide direct and clear research results for sustainable human development. However, most of the existing studies either require detailed data on energy consumption and its influencing factors, or rely on complex and time-consuming physical models that require detailed building and background environmental parameters and can usually only simulate individual buildings. How to mitigate the impacts of climate change on buildings energy requirements and guarantee high levels of energy efficiency in the building sector should be highly emphasized. These studies may be carried out at different scales (single building, building block, city and region scales), or by considering different elements (building, vegetation, and waterbody), by making use of multisource data and various methods.

(2) Previous studies have analyzed the interannual spatial-temporal changes in degree days during all-day periods in a certain region on the station or regional scale in both the past and the future. However, the vast majority of these studies used the degree days calculated based on the daily air temperature of all stations, ignoring the influences of relative humidity, solar radiation, population, etc., failed to indicate the inter-day changes in detail, and neglected the different mechanisms between the daytime or nighttime, among different seasons, under different environmental contexts, and in urban, suburban, and rural regions. Moreover, more effective methods should be introduced to more deeply analyze spatial-temporal changes more deeply, such as the Hurst exponent method, REOF method, geostatistical and landscape analysis, etc. Research on influencing factors of spatial-temporal changes in degree days was quite limited. It is urgent to deepen the study of more influencing factors of degree days in different time periods or environmental contexts, such as the remotely sensed indices of building, vegetation, water, bare soil, albedo, nighttime light intensity, etc.

(3) Some existing studies on the impacts of urban climate change on building heating and cooling energy consumption based on the degree days method were usually limited to comparing the differences in HDDs or CDDs between urban and suburban or rural stations for one city. In the future, these studies can be carried out to analyze the impacts of urbanization on HDDs, CDDs, and HDDs + CDDs for vast cities in different environment contexts at the station site, city, region, and global scales. These studies should use hourly or subhourly data, consider several key factors of degree days, including temperature, humidity, population, etc., and take into account the differences in urbanization impacts on degree days within the same city, between daytime and nighttime, among different seasons and years. A large number of previous studies on urban heat islands, the contribution of urbanization to temperature change, etc., can provide important theoretical and methodological references for analyzing the impacts of urbanization on degree days.

(4) The production and sharing of related datasets (not limited to weather datasets of the past and future) with high accuracy at high spatial and temporal resolutions are extremely important for future studies. This will require many multidisciplinary efforts and cross-disciplinary studies in the future.

(5) Not only are the impacts of climate change on the energy consumption of building heating and cooling considered, but further induced issues should also be emphasized, such as the emissions of carbon dioxide and air pollutants, decrease in the comfort level in human settlements, and increases in the disease risk among residents, etc.

(6) It is possible to solve the above issues by comprehensively utilizing multisources data and several methods. These data may include hourly or subhourly meteorological data from high-density stations, long-term historical observation data from stations, remote sensing data, and population data, etc. The necessary methods may involve station observation methods, geographic information technology, remote sensing method, machine learning method, etc.

## Figures and Tables

**Table 1:** Basic operations, advantages, and disadvantages of different methods used to estimate the impacts of climate change on the energy consumption of building heating and cooling.

Method Name	Basic Operations	Advantages	Disadvantages	Representative Studies
Statistical method	Establish the relationship between energy consumption and its influencing factors.	Estimate the specific amounts of influences of climate change on building heating and cooling energy consumption at a single building, city, or region scale.	⯎ Difficult to obtain the necessary actual building heating and cooling energy consumption records with a long historical span and the same spatial and temporal caliber for the corresponding driving factors; ⯎ Difficult to completely eliminate the interferences of non-climatic factors; ⯎ Low spatial and temporal resolution.	[6,14,22,24,26]
Physical model method	Use software to simulate the heating and cooling energy consumption of representative manual set or actual existing buildings under past, present, and future climate and building parameters.	Estimate the specific amounts of influences of climate change on building heating and cooling energy consumption of a single building in high temporal resolution under various scenarios.	⯎ Only suitable for a single building or local scale studies; ⯎ Require very detailed building and environmental parameters; ⯎ Require a large amount of computational time; ⯎ Require a high level of professional knowledge.	[29,30,31,32,33]
Comprehensive assessment method	Only choose some key parameters to roughly simulate or calculate the effects of climate change on building heating and cooling energy consumption.	Estimate the specific amounts of influences of climate change on building heating and cooling energy consumption at the year or month scale in regional or global scale.	⯎ Difficult to guarantee accuracy because unknown parameters are roughly and simply set to a fixed value; ⯎ Low spatial and temporal resolutions.	[7,34,35,36,37]
Combination method of the statistical and physical model methods	Establish the relationship between simulated energy consumption by physical model rather than actual consumption and the influencing factors.	Estimate the specific amounts of influences of climate change on building heating and cooling energy consumption in a single building without using actual energy consumption data.	⯎ Cannot avoid the inherent drawbacks in the physical model method.	[29,38,39,40]
Degree days method	Analyze the spatial-temporal evolution of degree days and their differences between at the urban and suburban or rural stations.	Simple, efficient and reliable to represent the impacts of climate change on the energy consumption of building heating and cooling.	⯎ Cannot obtain the specific amounts of building energy consumption affected by climate change; ⯎ Does not consider the effects of non-climatic factors in the vast majority of studies.	[41,42,43,44,45]

**Table 2:** Some appliance distributions and material properties of the buildings required in the physical model and sources [30].

Variable	Source
Building size (sqft)	Assessor DB ^1^
Building age	Assessor DB/RASS ^2^
Number of stories	RASS
Number of bedrooms	RASS
Presence of garage	RASS
Cooling technology and age	RASS
Heating technology and age	RASS
Window quality	RASS
Framing and foundation	Assessor handbook
Exterior finishes	Assessor handbook
Interior finishes	Assessor handbook
Ceiling fans	RASS
Temperature set point	RASS
Water heater technology and age	RASS

^1^ DB, Database; ^2^ RASS, Residential Appliance Saturation Survey.

**Table 3:** Studies on influencing factors of heating and cooling degree days.

Study Area	Degree Days Indices	Influencing Factors	Analysis Methods	References
Madison, Wisconsin USA	HDDs ^1^ and CDDs ^2^	Percent impervious surface coverage, lake effects, topographic relief	Linear and spatial regression	[41]
Bangladesh	HDDs and CDDs	Latitude, longitude, altitude, annual mean daily temperature, 10 large scale atmospheric circulation indices	Linear regression method, Pearson correlation, random forest (RF) model	[53]
Florence, Italy	CDDs	Impervious surfaces	Linear regression	[68]
Andalusia Autonomous Region, Spain	HDDs and CDDs	Elevation, distance to the sea	Semi-partial correlation	[79]
Xinjiang Province, China	HDDs and CDDs	Latitude, longitude, and altitude	Linear regression method	[80]

^1^ HDDs, heating degree days; ^2^ CDDs, cooling degree days.

**Table 4:** Percent of spatial variation explained by three influencing factors for spring 2012 to spring 2015 averages of heating and cooling degree days in Madison, Wisconsin, USA [41].

Parameter	Var. Explained (%)		
	Percent Impervious	Lake Proximity	Topographic Relief
HDDs ^1^	67	6	2
CDDs ^2^	75	3	2

^1^ HDDs, heating degree days; ^2^ CDDs, cooling degree days.

## Data Availability

No new data were created or analyzed in this study. Data sharing is not applicable to this article.
